# Intermammary ulcer in an elderly woman

**DOI:** 10.1097/JW9.0000000000000273

**Published:** 2026-06-26

**Authors:** Francisco Martins, José Carlos Cardoso, Joana Calvão

**Affiliations:** a Dermatology Department, University Hospital, Coimbra’s Local Health Unit, Coimbra, Portugal

**Keywords:** cutaneous adenocarcinoma, hormone receptors, hormonotherapy, skin neoplasms (MeSH), sweat gland neoplasms (MeSH), tumor (MeSH)

**What is known about this subject in regard to women and their families?**

- Distinguishing adnexal adenocarcinoma from cutaneous metastasis of breast carcinoma can be challenging in women because of overlapping histopathologic and immunohistochemical features.
- Management typically involves wide local excision, which may be associated with significant morbidity in older patients.

**What is new from this article as messages for women and their families?**

- This case underscores the diagnostic complexity of a hormone receptor-positive adnexal adenocarcinoma that initially mimicked metastatic breast carcinoma.
- It further demonstrates the successful use of hormone therapy with anastrozole as a nonsurgical treatment option, providing a less invasive therapeutic alternative for selected patients.

An 86-year-old woman presented with a painful intermammary lesion that had enlarged over 1 year. She denied fever or systemic symptoms.

Physical examination revealed an intermammary ulcer with elevated borders and an indurated base, measuring 5 × 4 cm (Fig. 1A). The lesion was firm and adherent to underlying tissues. Breast palpation was unremarkable, and no axillary or cervical lymph nodes were palpable.

**Fig. 1.**
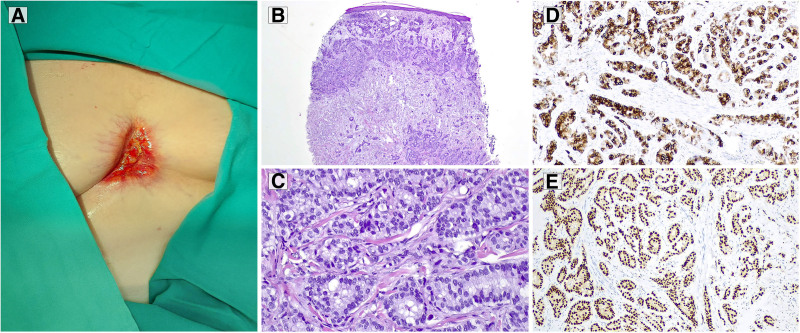
Adnexal adenocarcinoma, not otherwise specified (AA-NOS): clinical, histopathological, and immunohistochemical findings. (A) Clinical presentation showing a painful intermammary ulcer measuring 5 × 4 cm, with elevated borders and an indurated base, firmly adherent to underlying tissues. (B and C) Histopathology demonstrating a dermal infiltrate composed of trabeculae of neoplastic cells with abundant cytoplasm and pleomorphic nuclei, showing ductal differentiation (hematoxylin and eosin stain; original magnification ×10 in B and ×40 in C). (D) Immunohistochemical staining showing tumor cell positivity for cytokeratin 7 (CK7). (E) Strong and diffuse nuclear expression of estrogen receptors (ER) in tumor cells.

Histopathological analysis revealed a dermal infiltrate composed of trabeculae of neoplastic cells with abundant cytoplasm and pleomorphic nuclei, showing ductal differentiation (Fig. 1B and C). The tumor cells were positive for CK7 (Fig. 1D) and GATA3, with strong expression of progesterone (90%) and estrogen receptors (100%) (Fig. 1E). HER2 was negative. Ki-67 was 20%. CK20, TTF1, PAX8, and SATB2 were negative.

Positron emission tomography-computed tomography showed hypermetabolic thickening confined to the intermammary region, without other lesions. Breast magnetic resonance imaging was normal.

The differential diagnosis included basal cell carcinoma, cutaneous metastases from visceral adenocarcinomas, and adnexal adenocarcinomas. Basal cell carcinoma lacks ductal differentiation and hormone receptor expression; instead, the findings supported a diagnosis of adenocarcinoma and warranted exclusion of visceral metastasis.^1^ The tumor location and immunophenotype (CK7+, GATA3+, and ER+) raised suspicion for a breast origin, as breast carcinoma is the most common source of cutaneous metastases in women.^1,2^ Skin metastases from colorectal and lung adenocarcinomas were less likely, as they typically show positivity for SATB2 and TTF1, respectively.^1^

Given the absence of extracutaneous disease on breast magnetic resonance imaging and positron emission tomography-computed tomography, an adnexal adenocarcinoma was favored, as distinction from cutaneous metastasis cannot be established on histopathologic grounds alone. The absence of features of apocrine differentiation, namely decapitation secretion and abundant eosinophilic or granular cytoplasm, argued against apocrine carcinoma and supported a diagnosis of adnexal adenocarcinoma, not otherwise specified.^3^

After multidisciplinary discussion, given the patient’s age, lesion size, and diffuse ER positivity, primary hormone therapy with anastrozole (1 mg daily) was preferred over surgery. This treatment led to complete lesion resolution within 5 months (Fig. 2). At 2 years of follow-up, the patient remains disease-free. Maintenance anastrozole therapy will be continued, with ongoing clinical surveillance. Nutritional guidance emphasizing a calcium-rich diet was provided to reduce adverse effects.

**Fig. 2.**
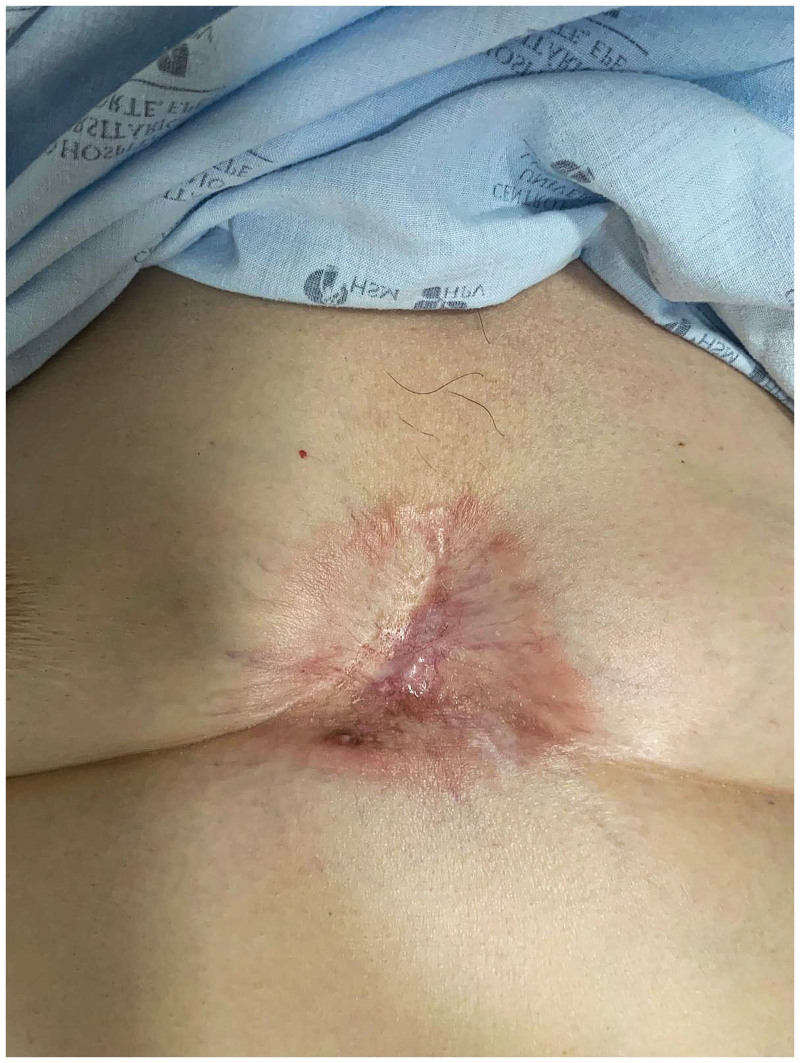
Therapeutic response of adnexal adenocarcinoma, not otherwise specified (AA-NOS), to anastrozole. Complete clinical resolution of the intermammary ulcer after 5 months of treatment with anastrozole (1 mg daily), with residual cicatricial changes and no evidence of active disease. At 2 years of follow-up, the patient remains disease-free.

Adnexal adenocarcinoma, not otherwise specified, is a rare malignant adnexal tumor characterized by ductal differentiation without definitive eccrine or apocrine features.^3^ Because of its rarity, management is individualized and largely extrapolated from experience with other malignant adnexal tumors. Surgical excision remains the main treatment for localized disease, while radiotherapy and chemotherapy have been used in selected cases; however, many tumors appear relatively resistant, and chemotherapy is generally reserved for metastatic disease.^3^ Antiestrogen therapy has also been reported as a therapeutic option in estrogen receptor-positive adnexal tumors.^4^

Malignant adnexal tumors may recur or metastasize, and distant and nodal metastases have been identified as the main predictors of poorer survival.^3,5^

In this case, anastrozole was selected over tamoxifen due to the patient’s postmenopausal status, providing more effective estrogen suppression with fewer risks. Despite the limited number of reported cases, hormone-responsive adnexal tumors appear to have a favorable prognosis,^4^ particularly in the absence of systemic spread.

## Conflicts of interest

None.

## Funding

None.

## Study approval

N/A.

## Author contributions

FM: Co-designed the overall structure of the paper, conducted the literature review, and wrote the paper. JCC: Performed the histopathological differential diagnosis and contributed to manuscript revision. JC: Co-designed the overall structure of the paper and critically reviewed the final version.

## Data availability

Relevant clinical data, histopathologic images, immunohistochemical results, and complementary diagnostic examinations supporting the findings of this study are available from the corresponding author upon request.

## Ethics statement

Informed consent was obtained, and all procedures in this work were in accordance with current ethical standards.
